# Picodroplet partitioned whole genome amplification of low biomass samples preserves genomic diversity for metagenomic analysis

**DOI:** 10.1186/s40168-016-0197-7

**Published:** 2016-10-06

**Authors:** Maria Hammond, Felix Homa, Helene Andersson-Svahn, Thijs J. G. Ettema, Haakan N. Joensson

**Affiliations:** 1 Department of Cell and Molecular Biology, Science for Life Laboratory, Uppsala University, Uppsala, Sweden; 2 Division of Proteomics and Nanobiotechnology, Science for Life Laboratory, Royal Institute of Technology (KTH), Stockholm, Sweden

**Keywords:** Whole genome amplification, Multiple displacement amplification, Metagenomics, Droplet microfluidics, Amplification bias

## Abstract

**Background:**

Whole genome amplification (WGA) is a challenging, key step in metagenomic studies of samples containing minute amounts of DNA, such as samples from low biomass environments. It is well known that multiple displacement amplification (MDA), the most commonly used WGA method for microbial samples, skews the genomic representation in the sample. We have combined MDA with droplet microfluidics to perform the reaction in a homogeneous emulsion. Each droplet in this emulsion can be considered an individual reaction chamber, allowing partitioning of the MDA reaction into millions of parallel reactions with only one or very few template molecules per droplet.

**Results:**

As a proof-of-concept, we amplified genomic DNA from a synthetic metagenome by MDA either in one bulk reaction or in emulsion and found that after sequencing, the species distribution was better preserved and the coverage depth was more evenly distributed across the genomes when the MDA reaction had been performed in emulsion.

**Conclusions:**

Partitioning MDA reactions into millions of reactions by droplet microfluidics is a straightforward way to improve the uniformity of MDA reactions for amplifying complex samples with limited amounts of DNA.

**Electronic supplementary material:**

The online version of this article (doi:10.1186/s40168-016-0197-7) contains supplementary material, which is available to authorized users.

## Background

Most of the world’s microbial diversity remains unknown [1, 2]. With improving sequencing capacities at declining costs, the actual sequencing is no longer the major bottleneck for obtaining genome sequence data of unknown, non-culturable microbial species, the so-called microbial dark matter. Apart from data analysis and interpretation, a major challenge in metagenomic studies is obtaining high-quality sequencing libraries from environmental samples that only contain minute amounts of DNA. Commercially available library preparation kits recommend using nanograms of input DNA, i.e., approximately one million cells, at a minimum. Such amounts may not be available for low biomass environments [3], mini-metagenomes [4], and single-cell genomes [2, 5].

Multiple displacement amplification (MDA) [6] is the most commonly used method for whole genome amplification (WGA) of small amounts of microbial genomic DNA due to the high yield and low error rate of the *Phi29* polymerase employed. However, the MDA reaction has drawbacks that include biased amplification of different genomic regions resulting in uneven coverage depths of these regions. For metagenome samples, this bias results in a skewed representation of the relative abundance of species, even at relatively high concentrations of input material (nanograms) [7–11]. In addition, formation of chimeras—noncontiguous sequences joined together during the amplification—has been reported for MDA, potentially confounding the sequencing results [12]. The skewed relative representation of different genomic regions and presence of chimera make the assembly of complete and accurate genomes from samples amplified by MDA prior to library preparation more difficult than that of corresponding samples where greater amounts of sample DNA allow direct library preparation without prior amplification [13]. Sequencing libraries can be prepared from lower input amounts than those recommended [14–16], but this is also associated with increased bias, e.g., overrepresentation of GC-rich sequences [16].

A monodisperse emulsion of millions of picoliter-sized droplets can easily be generated in a droplet microfluidics device where the aqueous reaction mixture is partitioned into droplets in a fluorinated oil with added surfactant by flow-focusing [17, 18]. Each droplet thus functions as an isolated reaction chamber, compartmentalizing the reaction into multiple parallel reactions. It was recently reported that partitioning the MDA reaction into millions of droplets rather than a single microliter scale reaction improves the coverage, both in coverage breadth (the proportion of the genome being sequenced) and in evenness of the coverage depth across the genome, when sequencing single human [19] or *E. coli* [20, 21] cells. Here, we report how the same strategy can be used to improve MDA of limited amounts of DNA in mixed species samples.

## Results

For the purpose of this study, we prepared a synthetic metagenome by mixing genomic DNA from five different species, *Terriglobus roseus*, *Coraliomargarita akajimensis*, *Pseudomonas stutzeri*, *Phaeobacter inhibens*, and *Geodermatophilus obscurus,* at different ratios (Additional file 1: Table S1). We diluted it to concentrations well below the recommended input concentrations for commercial library preparation kits (0.16–4 pg/μl), amplified it, prepared sequencing libraries, and sequenced with Illumina MiSeq 2 × 300 bp (Additional file 1: Table S2). The aimed for relative abundances of genomic DNA from different species are only relative estimates. To assess the performance of the amplification in this study, we sequenced libraries from the unamplified sample to use as ground truth. We used two independently pooled mock community samples that display slight variations in terms of relative abundance. Relative species abundance is thus not compared between the two independently pooled synthetic metagenomes, but data demonstrating relative species abundance for the samples amplified at 1 pg/μl and their corresponding unamplified control are presented in the supplementary material.

### Multiple displacement amplification in emulsion

**Fig. 1:**
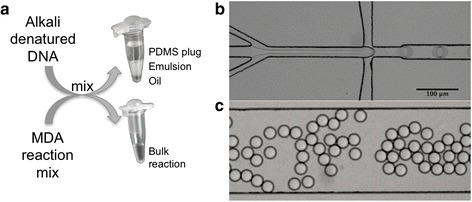
Illustration of the method. **a** DNA is denatured with alkaline solution and neutralized, and the MDA reaction mix is prepared. Aliquots of these solutions are mixed either directly for a bulk reaction in a PCR tube or in a microfluidic chip **b** to generate an emulsion with **c** homogeneous picoliter-sized droplets. The generated emulsion is collected in a PCR tube plugged by a PDMS plug to allow maintained droplet stability during long-term incubation


To set up the MDA reactions in emulsion, the template DNA is first denatured with alkaline solution and neutralized. The denatured DNA is loaded into a microfluidic chip where it is mixed with MDA reaction mix immediately prior to droplet generation (Fig. 1, Additional file 2). When the amplification is terminated, the emulsion is destabilized by addition of an emulsion breaker. This separates the aqueous phase containing the MDA products from the oil due to the large density difference, allowing the MDA products to be easily recovered by pipetting. The MDA products from the emulsion can then be treated in the same way as MDA products that were generated in a standard bulk MDA reaction.

Additional file 2Video of droplet generation. (M4V 738 kb)


### Reduced amplification of contamination and primer-derived artifacts

**Fig. 2:**
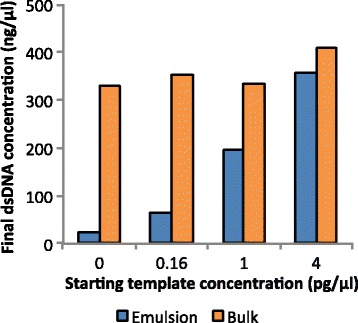
Amplification yields from MDA reactions in emulsion and standard bulk reactions from different starting template concentrations. Final double stranded (ds) DNA concentrations were measured after breaking the emulsion and collecting the aqueous phase from all droplets of the reaction

The yields from MDA performed in emulsion with all droplets containing template were well above 100 ng/μl, similar to the standard bulk reaction (Fig. 2). With input concentrations lower than one molecule per droplet on average the yields decrease, in contrast to the MDA reactions performed in bulk where the final DNA concentrations were well above 100 ng/μl independent of template starting concentrations. The negative control without any added template DNA also yielded similar amounts of amplified DNA in the bulk reaction while the yield from the no template negative control was substantially lower after MDA in emulsion compared to in bulk.

We prepared libraries and sequenced the no template negative control samples, aligned the reads to NCBI nucleotide database, and found that a majority of the reads did not map to any known sequences, indicating that those were primer dimer-derived artifacts from the MDA. The remaining reads could be mapped to expected contaminants such as *Homo sapiens*, commensal skin bacteria, and other previously described contaminants of molecular biology kits, mainly from bacterial genus *Herbaspirillum* [22]. The ratio of sequenced reads without hits and identified contaminants from the emulsion-amplified sample was similar to the bulk-amplified sample. This indicates that primer dimer artifacts are formed and that contamination is present in emulsion too. Yet, the contamination and primer dimer artifacts are limited to only a small fraction of the droplets and hence never dominate the entire MDA reaction volume.

### Quality of sequenced reads

After sequencing the amplified DNA and a sample of the unamplified synthetic metagenome, more than 95 % of the reads that pass the quality control map to the five reference genomes (Table 1). More than 90 % of the reads are reported by Samtools as properly paired, i.e., both reads in a read pair map to the same genome in the expected orientation and distance from each other. For the unamplified control, more than 99 % of the reads are properly paired, indicating some chimera formation during the MDA reaction both in bulk and in emulsion.

**Table 1:** Percentage of reads in each sample mapping to any of the five reference genomes

MDA in	MDA input conc. (pg/μl)	% mapped reads	% properly paired mapped reads
Emulsion	4	99.80	91.19
Emulsion	1	99.02	95.51
Emulsion	0.16	96.89	94.16
Bulk	4	99.64	93.96
Bulk	1	99.61	93.44
Bulk	0.16	98.56	92.62
Unamplified	n.a.	99.58	99.27
Unamplified2	n.a.	99.43	99.17

### Better maintained species distribution

**Fig. 3:**
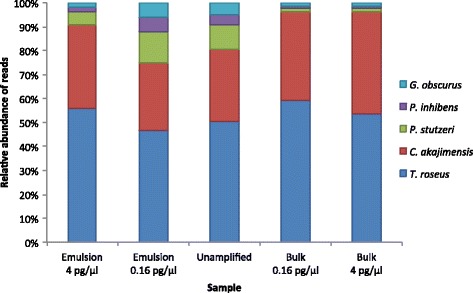
Proportion of properly paired reads mapping to respective reference genome. Absolute numbers of reads are listed in Additional file 1: Table S3

It is known that MDA can change the species distribution after amplification of mixed species samples [7–11]. We observe an extensive reduction in representation of the three most rare species in the samples amplified in bulk, while their representation is still similar to the unamplified sample after amplification in emulsion (Fig. 3, Additional file 1: Figure S1, Table S3).

**Fig. 4:**
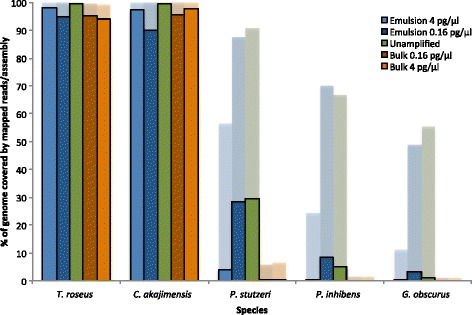
Coverage breadth of mapped reads and assembled contigs. *Semi-transparent bars* at the back show the percentage of the genomes that are covered at least once when reads subsampled to include the same total amount of data for all samples were mapped to the reference genomes. *Dense bars* at the front show the proportion of the genomes that are covered with de novo assembled contigs from the same data set

In order to gain insight into how much of the genomic diversity present in the original sample we manage to sequence, we evaluated how much of each genome was covered at least once when using equal amounts of data for each sample. We subsampled the data to include the same amount of data for each sample and re-mapped that data individually to each of the five reference genomes and analyzed the coverage (Additional file 1: Table S4). As expected, considering the larger amount of reads from the rarer genomes when the MDA was performed in emulsion, much larger proportions of the three more rare genomes were covered when MDA had been performed in emulsion compared to in bulk (Fig. 4, Additional file 1: Figure S2, Table S4). From the samples amplified in emulsion, we sequenced a greater proportion of the more rare genomes when supplying the lower input concentration, since for each further sample dilution a greater fraction of the DNA containing droplets contain only a single template molecule so that more template molecules get the chance to reach saturation in the amplification without inhibition from other templates being amplified more rapidly. The samples amplified in bulk did not exhibit a similar trend.

### More even coverage depth across the genome

**Fig. 5:**
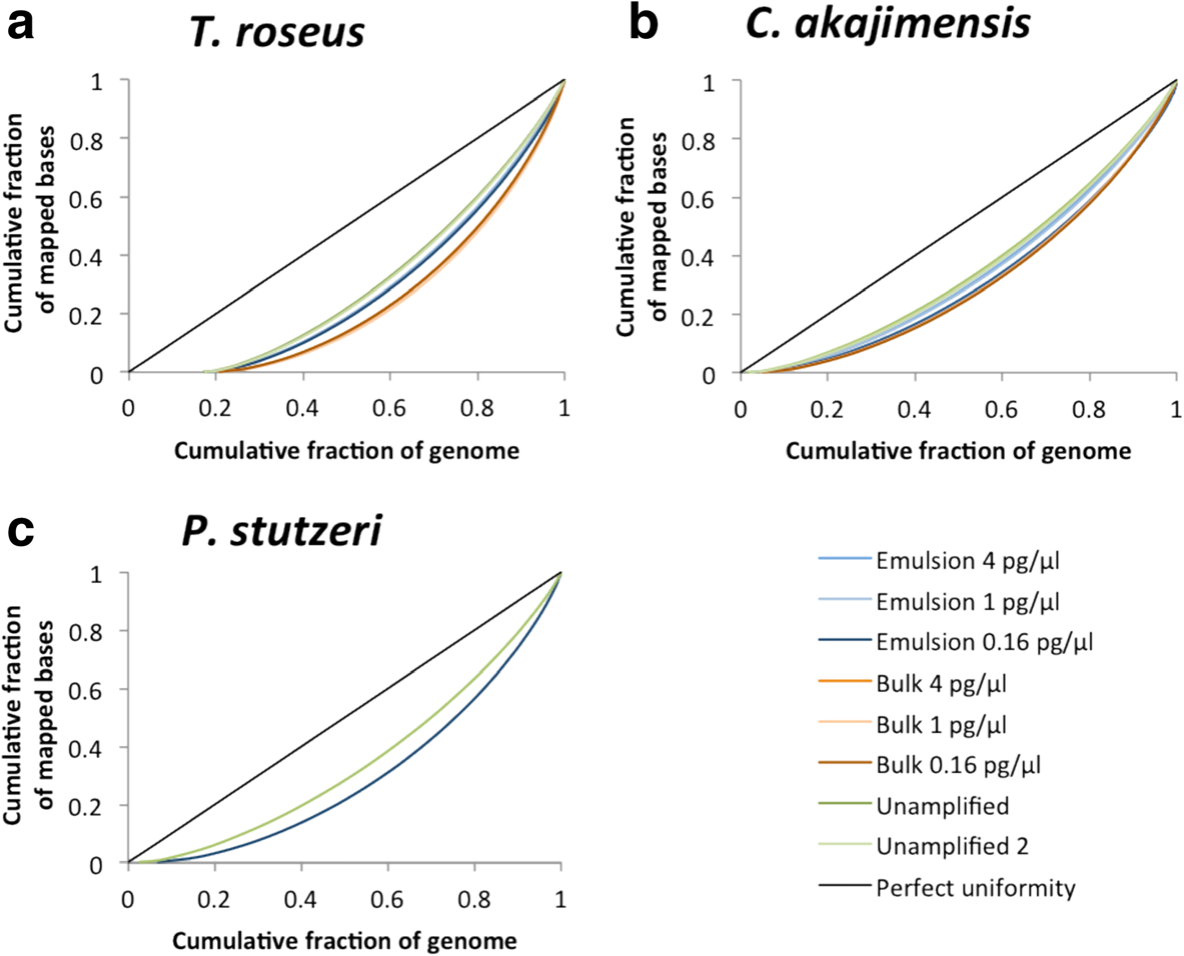
Lorenz curves showing the cumulative fraction of mapped bases plotted as a function of the cumulative fraction of the genome that is covered at least once for **a** *T. roseus*, **b** *C. akajimensis*, and **c** *P. stutzeri*. Prior to analysis, the data was subsampled to include the same amount of data, corresponding to an average 5× coverage depth for each respective genome, for all samples

To examine the distribution of the coverage depth across each genome, we subsampled the data to have on average 5× coverage of mapped properly paired reads to each respective genome (Additional file 1: Tables S5–S7). This subsampling was only possible for the two most abundant species *T. roseus* and *C. akajimensis* for all samples and for *P. stutzeri* for the unamplified sample and the sample amplified in emulsion from the lowest input DNA concentration. We re-mapped the subsampled reads to each respective genome (Additional file 1: Figures S3–S5) and calculated the coefficient of variation (CV) of the coverage depth for each position in the genome (Table 2). We also plotted the data as Lorenz curves (Fig. 5) and calculated the Gini coefficients (Table 2). Both the CVs and the Gini coefficients indicate that MDA in emulsion amplifies the genomes more evenly compared to MDA in bulk.

**Table 2:** Characteristics of coverage depth for each genome

Species	MDA in	MDA input conc. (pg/μl)	% of genome covered at least 1×	CV %	Gini coefficient
*T. roseus*	Emulsion	4	81.79	78.05	0.43
	Emulsion	1	81.92	78.29	0.43
	Emulsion	0.16	81.79	83.03	0.44
	Bulk	4	79.46	96.17	0.51
	Bulk	1	78.85	100.82	0.52
	Bulk	0.16	79.29	95.97	0.51
	Unamplified	n.a.	82.88	69.37	0.39
	Unamplified2	n.a.	82.59	70.76	0.4
*C. akajimensis*	Emulsion	4	97.55	56.89	0.31
	Emulsion	1	97.51	59.19	0.32
	Emulsion	0.16	96.29	77.05	0.36
	Bulk	4	95.29	69.59	0.37
	Bulk	1	95.29	70.61	0.37
	Bulk	0.16	95.06	71.18	0.38
	Unamplified	n.a.	98.37	50.74	0.28
	Unamplified2	n.a.	98.01	53.26	0.3
*P. stutzeri*	Emulsion	0.16	93.01	75.62	0.4
	Unamplified	n.a.	97.41	55.22	0.3

There is a region in the *T. roseus* with very low coverage in all samples including the unamplified control (Additional file 1: Figure S3). Upon closer inspection of the reference genome, we noticed that this is a 460-kb duplicated region, possibly caused by an assembly error in the *T. roseus* reference genome. Reads that mapped to the reference genome more than once were excluded during the subsampling to on average 5× coverage depth, meaning that hardly any reads mapping to the duplicated region in the *T. roseus* reference genome were included in this analysis. This explains why none of the samples cover more than 83 % of this genome.

### Longer de novo assemblies

We also performed de novo assemblies from the same total amount of data for all samples individually. It is clear that total assembly sizes are larger from the samples amplified in emulsion compared to in bulk and especially from the sample with the lowest input concentration (Table 3, Additional file 1: Table S8). We could assemble 90 % or more of the genomes of the two most abundant species, while the three more rare species are partially covered, only by contigs from the unamplified sample and the samples amplified in emulsion, again with a substantially higher percentage of the genome covered from the sample amplified in emulsion from the lowest template concentration (Fig. 4, Additional file 1: Figure S2, Table S9). We found that assembled contigs are shorter in the samples with lower template concentrations (Additional file 1: Table S9). These findings are consistent with the findings by Bowers et al. [16] where they generated sequencing libraries from much smaller quantities of DNA than recommended and assembled shorter contigs with decreasing amounts of input DNA.

**Table 3:** Basic statistics from de novo assemblies

MDA in	MDA input conc. (pg/μl)	Total length	# contigs	Largest alignment	GC%
Emulsion	4	8482128	673	160736	57.42
Emulsion	0.16	9700908	2009	96006	58.48
Bulk	4	8124432	783	216284	57.26
Bulk	0.16	8099511	843	104196	57.34
Unamplified	n.a.	9992266	1121	242057	58.21

Interestingly, we assembled *T. roseus* genomes that according to MetaQUAST analyses are close to complete for all samples, but still the total length of the assemblies are substantially shorter than the reference sequence (Additional file 1: Table S9). When we align the assembled contigs from the unamplified sample to the reference sequence, one of the contigs map twice with 99.5 % identity, explaining why the fraction of the genome covered by contigs is close to 100 %, despite the shorter total length. This is the same duplicated region that is poorly covered after subsampling to 5× average coverage of the *T. roseus* genome.

## Discussion

We have demonstrated how partitioning of the template DNA molecules of a mixed species sample into separated parallel MDA reactions better maintains the species distribution of the original sample. In the demonstrated experiments, the species distribution is best preserved with a lower template concentration, but a lower template input does, as expected, have a negative impact on the length of the contigs in de novo assembly. To prepare sequencing libraries that optimally represent the original diversity of the sample, the highest possible starting amount of template DNA should be used. The protocol should then be optimized to include as much as possible of the original sample but still with the template molecules distributed to single or very few copies per droplet. This can be achieved either by increasing the total volume of MDA reaction mix that is emulsified or by decreasing the size of each droplet.

The strategy of partitioning a complex, multi-target reaction into millions of low-complexity, single or few-copy target reactions for a more uniform total amplification is not limited to MDA. It should also be valid for other methods, such as other WGA methods, the PCR enrichment step in library preparations or any reaction where there is a risk that only a fraction of the original molecules in a diverse sample will saturate the amplification. Performing the PCR enrichment step of a sequencing library preparation in emulsion could thus be another way to improve metagenome analysis from low biomass samples. In the presented experiments, droplets were generated by an in-house built system, but this could also have been achieved by using commercially available droplet generation systems.

## Conclusions

We demonstrate that by partitioning the MDA reaction in an emulsion of millions of picoliter-sized droplet reaction chambers, we amplify a mixed microbial species sample more uniformly than when the reaction is performed in a single bulk reaction. Since it is the same enzymatic reaction that is used, we maintain all desirable characteristics of the MDA reaction, such as proof reading for high fidelity, and high yield, but limit the bias in the amplification and the impact of contamination and primer derived artifacts. Our findings suggest that quantitative studies of metagenomes from low biomass environments, where it is not possible to extract the amounts of DNA required for downstream analysis, can be achieved after MDA in emulsion.

## Methods

### Genomic DNA

Purified genomic DNA from *T. roseus* (DSM 18391), *C. akajimensis* (DSM 45221), *P. stutzeri* (DSM 4166), *P. inhibens* (DSM 17395), and *G. obscurus* (DSM 43160) were purchased from DSMZ. Concentrations were determined by NanoDrop 2000 (Thermo Scientific) absorbance measurements at 260 nm and Qubit double-stranded DNA (dsDNA) kit (ThermoFisher Scientific).

### Microfluidic device fabrication and operation

A microfluidic chip with one inlet for the fluorinated oil with surfactants and two inlets for DNA solution and MDA reaction mix, respectively, was fabricated in polydimethylsiloxane (PDMS) and glass by soft lithography [23] as previously described [24]. The design is presented in Additional file 1: Figure S6. The channel depth is 25 μm and the nozzle width, where the aqueous phase meets the oil, is also 25 μm. We generated droplets with a volume of approximately 10 pl (26 μm in diameter) by injecting the two aqueous solutions at flow rates of 100 μl/h each and the oil (Novec HFE-7500 fluorinated oil, 3 M) with 1 % (*w*/*w*) EA surfactant droplet stabilizer (RainDance Technologies) at 1000 μl/h. The aqueous solutions were injected from 1-ml plastic syringes (BD Plastipak) and the oil from a Gastight 2.5-ml glass syringe (Hamilton) connected to the chip via polyetheretherketone (PEEK) tubing (Zeus). Flows were controlled by neMESYS dosing units and software (Cetoni GmbH). The generated emulsion was passively collected via tubing into a 0.2-ml PCR tube pre-filled with HFE-7500 with 1 % EA and plugged by a PDMS plug (see photo in Fig. 1a) as previously described [25]. Droplet generation was monitored and imaged using an inverted microscope (Olympus IX51) with a CCD camera (Allied Vision).

### Multiple displacement amplification

DNA was denatured by mixing the DNA diluted in milliQ water 1:1 with 50 mM KOH (Sigma Aldrich) and incubating for 3 min at room temperature (RT). The denatured DNA was the neutralized by adding an equal volume of Tris-HCl (80 mM, pH4; Sigma Aldrich). RepliPHI Phi29 Reagent Kit (Epicenter) supplemented with Exo-Resistant Random Primer (ThermoFisher Scientific) was used for the MDA reaction. A 2× MDA mastermix (2× reaction buffer, 2 mM dNTP, 50 μM primer, 4 U/μl Phi29, 8 mM DTT and 5 % DMSO) was prepared. The denatured and neutralized DNA and the 2× MDA mastermix were mixed at equal volumes by pipetting for a bulk reaction in tube or in the microfluidic chip as described above for emulsion generation. Reactions were incubated for 12 h at 30 °C. The polymerase was then inactivated at 65 °C for 10 min.

After incubation, the emulsion was broken by adding 5 μl 1H, 1H, 2H, 2H, Perfluoro-1-octanol (Sigma Aldrich), vortexing, and centrifuging briefly until the emulsion separated into one aqueous and one oil phase. If the emulsion did not break, the emulsion breaking procedure was repeated. The supernatant (aqueous phase) was collected by pipetting and could then be treated like the MDA products from the bulk reactions. The concentrations of MDA products were quantified with Qubit dsDNA kit (ThermoFisher Scientific) or Quant-iT PicoGreen dsDNA assay (ThermoFisher Scientific).

### Library preparation and sequencing

Sequencing libraries were prepared with Nextera XT Library Prep Kit (Illumina) according to manufacturer’s instructions for 2 × 300 runs on MiSeq, except input DNA concentrations were 0.4 ng/μl (2 ng in total) in order to increase the insert size of the sequencing libraries. Nextera Index Kit (Illumina) was used to barcode individual samples. Library concentrations were determined by Quant-iT PicoGreen dsDNA assay before pooling libraries with different index barcodes. Samples were sequenced with 2 × 300 runs on a MiSeq instrument (Illumina).

### Data analysis of sequenced libraries

The reads from the sequenced libraries were quality controlled and trimmed using Trimmomatic [26] to remove Nextera adapters and low quality data (requiring quality of 12 for sliding window of four nucleotides, minimal read length of 50 bp). Reads from negative controls (the MDA reactions without added template DNA) were aligned against NCBI nucleotide database using BLAST (standalone BLAST+ package version 2.2.30) [27]. Reads from positive samples, where DNA from the pooled mock communities had been added, were aligned to the reference genomes with BWA-MEM using default settings [28]. Mapping statistics were generated using the Flagstat module of Samtools 1.2 [29]. BEDtools 2.23.0 [30] was used to assess the coverage across the genomes.

To allow comparisons of the different libraries, we used the previously generated mapping files to subsample the data to include the same amount of data from each library for further analysis. We first removed all reads that were not paired in sequencing. Then, we subsampled the data to include, for each sample, the number of reads needed to include the same amount of data for all samples.

We also subsampled, for each sample, the number of reads that mapped in proper pairs to a single genome to include data corresponding to an average 5× coverage depth for that genome, in order to allow comparisons of the coverage depth across that genome. Prior to this subsampling, we filtered all BAM files to remove reads that mapped to more than one location in the genomes (reads with mapping quality of 0 according to BWA BAM specifications). Lorenz curves were prepared where the cumulative fraction of mapped bases was plotted as a function of the cumulative fraction of the genome that is covered at least once. This is a way to illustrate the uniformity of the coverage depth across the genome where a perfectly straight line on the diagonal would represent perfect uniformity where all bases of the genome were covered with the exact same number of sequenced reads. Gini coefficients were calculated as the area between the curve representing perfect uniformity and the curve of each sample in the Lorenz plots, using Riemann middle sum to approximate the areas under the curves. Coefficients of variation (CVs) were calculated as the standard deviation of the coverage depth for each position in the genome divided by the mean coverage depth across the entire genome.

De novo assemblies were performed with IDBA-UD 1.1.2 [31]. The quality of each of the assemblies was evaluated with MetaQUAST 3.1 [32] provided all five reference genomes.

## Additional files

Additional file 1:
**Figures S1–S6** and **Tables S1–S9**. (PDF 792 kb)

## References

[1] Wu D, Hugenholtz P, Mavromatis K, Pukall R, Dalin E, Ivanova NN (2009). A phylogeny-driven genomic encyclopaedia of bacteria and archaea. Nature.

[2] Rinke C, Schwientek P, Sczyrba A, Ivanova NN, Anderson IJ, Cheng J-F (2013). Insights into the phylogeny and coding potential of microbial dark matter. Nature.

[3] Gonzalez JM, Portillo MC, Saiz-Jimenez C (2005). Multiple displacement amplification as a pre-polymerase chain reaction (pre-PCR) to process difficult to amplify samples and low copy number sequences from natural environments. Environ Microbiol.

[4] McLean JS, Lombardo M-J, Badger JH, Edlund A, Novotny M, Yee-Greenbaum J (2013). Candidate phylum TM6 genome recovered from a hospital sink biofilm provides genomic insights into this uncultivated phylum. Proc Natl Acad Sci.

[5] Raghunathan A, Ferguson HR, Bornarth CJ, Song W, Driscoll M, Lasken RS (2005). Genomic DNA amplification from a single bacterium. Appl Environ Microbiol.

[6] Dean FB, Nelson JR, Giesler TL, Lasken RS (2001). Rapid amplification of plasmid and phage DNA using Phi29 DNA polymerase and multiply-primed rolling circle amplification. Genome Res.

[7] Binga EK, Lasken RS, Neufeld JD (2008). Something from (almost) nothing: the impact of multiple displacement amplification on microbial ecology. ISME J.

[8] Yilmaz S, Allgaier M, Hugenholtz P (2010). Multiple displacement amplification compromises quantitative analysis of metagenomes. Nat Methods.

[9] Direito SOL, Zaura E, Little M, Ehrenfreund P, Röling WFM (2014). Systematic evaluation of bias in microbial community profiles induced by whole genome amplification. Environ Microbiol.

[10] Marine R, McCarren C, Vorrasane V, Nasko D, Crowgey E, Polson SW (2014). Caught in the middle with multiple displacement amplification: the myth of pooling for avoiding multiple displacement amplification bias in a metagenome. Microbiome.

[11] Probst AJ, Weinmaier T, DeSantis TZ, Santo Domingo JW, Ashbolt N (2015). New perspectives on microbial community distortion after whole-genome amplification. PLoS One.

[12] Lasken RS, Stockwell TB (2007). Mechanism of chimera formation during the multiple displacement amplification reaction. BMC Biotechnol.

[13] Chitsaz H, Yee-Greenbaum JL, Tesler G, Lombardo M-J, Dupont CL, Badger JH (2011). Efficient de novo assembly of single-cell bacterial genomes from short-read data sets. Nat Biotechnol.

[14] Adey A, Morrison HG, Asan, Xun X, Kitzman JO, Turner EH (2010). Rapid, low-input, low-bias construction of shotgun fragment libraries by high-density in vitro transposition. Genome Biol.

[15] Chafee M, Maignien L, Simmons SL (2015). The effects of variable sample biomass on comparative metagenomics. Environ Microbiol.

[16] Bowers RM, Clum A, Tice H, Lim J, Singh K, Ciobanu D (2015). Impact of library preparation protocols and template quantity on the metagenomic reconstruction of a mock microbial community. BMC Genomics.

[17] Umbanhowar PB, Prasad V, Weitz DA (2000). Monodisperse emulsion generation via drop break off in a coflowing stream. Langmuir.

[18] Anna SL, Bontoux N, Stone HA (2003). Formation of dispersions using “flow focusing” in microchannels. Appl Phys Lett.

[19] Fu Y, Li C, Lu S, Zhou W, Tang F, Xie XS (2015). Uniform and accurate single-cell sequencing based on emulsion whole-genome amplification. Proc Natl Acad Sci.

[20] Nishikawa Y, Hosokawa M, Maruyama T, Yamagishi K, Mori T, Takeyama H (2015). Monodisperse picoliter droplets for Low-bias and contamination-free reactions in single-cell whole genome amplification. PLoS One.

[21] Sidore AM, Lan F, Lim SW, Abate AR. Enhanced sequencing coverage with digital droplet multiple displacement amplification. Nucleic Acids Res. 2016;44:e66. 10.1093/nar/gkv1493 PMC4838355 26704978

[22] Salter SJ, Cox MJ, Turek EM, Calus ST, Cookson WO, Moffatt MF (2014). Reagent and laboratory contamination can critically impact sequence-based microbiome analyses. BMC Biol.

[23] Xia Y, Whitesides GM (1998). Soft lithography. Angew Chem Int Ed.

[24] Sjostrom SL, Bai Y, Huang M, Liu Z, Nielsen J, Joensson HN (2014). High-throughput screening for industrial enzyme production hosts by droplet microfluidics. Lab Chip.

[25] Pekin D, Skhiri Y, Baret J-C, Le Corre D, Mazutis L, Salem CB (2011). Quantitative and sensitive detection of rare mutations using droplet-based microfluidics. Lab Chip.

[26] Bolger AM, Lohse M, Usadel B (2014). Trimmomatic: a flexible trimmer for Illumina sequence data. Bioinformatics.

[27] Altschul SF, Gish W, Miller W, Myers EW, Lipman DJ (1990). Basic local alignment search tool. J Mol Biol.

[28] Li H. Aligning sequence reads, clone sequences and assembly contigs with BWA-MEM. ArXiv1303.3997 Q-Bio. 2013.

[29] Li H, Handsaker B, Wysoker A, Fennell T, Ruan J, Homer N (2009). The sequence alignment/Map format and SAMtools. Bioinforma Oxf Engl.

[30] Quinlan AR, Hall IM (2010). BEDTools: a flexible suite of utilities for comparing genomic features. Bioinformatics Oxf Engl.

[31] Peng Y, Leung HCM, Yiu SM, Chin FYL. IDBA—a practical iterative de Bruijn graph de novo assembler. In: Berger B, editor. Res. Comput. Mol. Biol. Springer Berlin Heidelberg; 2010. p. 426–40

[32] Mikheenko A, Saveliev V, Gurevich A. MetaQUAST: evaluation of metagenome assemblies. Bioinformatics. 2016;32:1088–1090. 10.1093/bioinformatics/btv697 26614127

