# Supplementary material for: Picodroplet partitioned whole genome amplification of low biomass samples preserves genomic diversity for metagenomic analysis
Source: Microbiome. 2016 Oct 6;4:52. doi: 10.1186/s40168-016-0197-7 (PMC5054601; doi:10.1186/s40168-016-0197-7)
Supplement: Additional file 1: — Figures S1–S6 and Tables S1–S9. (PDF 792 kb) [file 40168_2016_197_MOESM1_ESM.pdf]

Additional file 1

List of content

Fig. S1. Proportion of properly paired reads mapping to respective reference genome
Fig. S2. Coverage breadth of mapped reads and assembled contigs
Fig. S3. Coverage depth across *T. roseus* genome
Fig. S4. Coverage depth across *C. akajimensis* genome
Fig. S5. Coverage depth across *P. stutzeri* genome
Fig. S6. Design of the microfluidic circuit used for droplet generation
Table S1. Genomic DNA pooled for synthetic metagenome
Table S2. Number of sequenced reads for each sample before and after QC trimming
Table S3. Number and percentage of reads mapping to the different species
Table S4. Statistics for reads mapped to all five reference genomes
Table S5. Number of reads and amount of data after subsampling to average 5x coverage of reads mapping to *T. roseus*
Table S6. Number of reads and amount of data after subsampling to average 5x coverage of reads mapping to *C. akajimensis*
Table S7. Number of reads and amount of data after subsampling to average 5x coverage of reads mapping to *P. stutzeri*
Table S8. Basic statistics for *de novo* assemblies
Table S9. Basic statistics for mapping contigs from MetaQUAST analysis

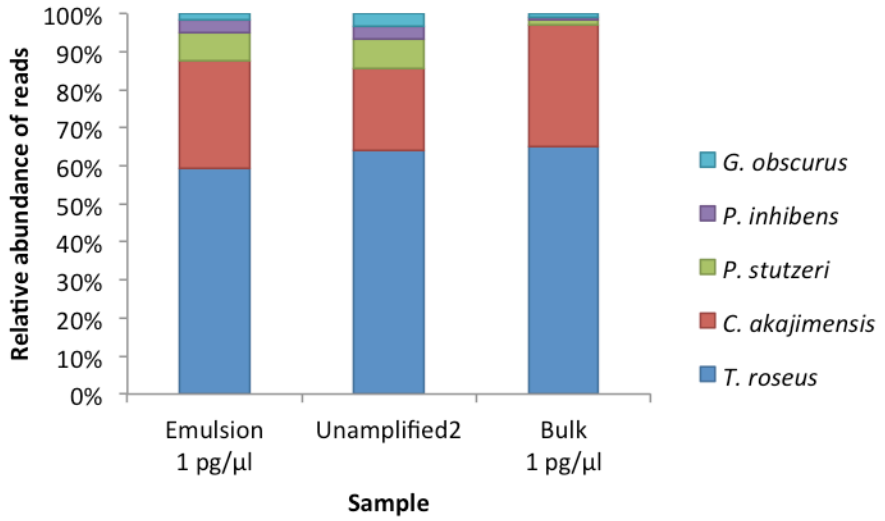

Fig. S1. Proportion of properly paired reads mapping to respective reference genome. Absolute numbers of reads are listed in Additional file 1: Table S3.

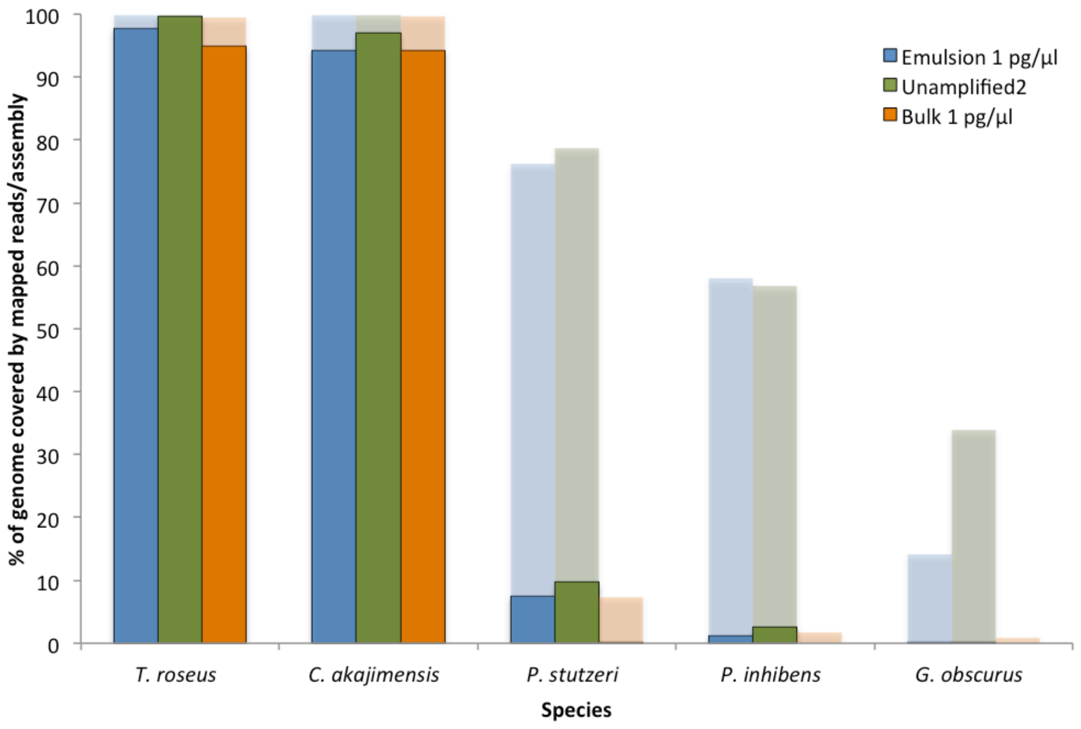

Fig. S2. Coverage breadth of mapped reads and assembled contigs. Semi-transparent bars in the back show the percentage of the genomes that are covered at least once when reads subsampled to include the same total amount of data for all samples were mapped to the reference genome. Dense bars in the front show the proportion of the genomes that are covered with *de novo* assembled contigs from the same data set.

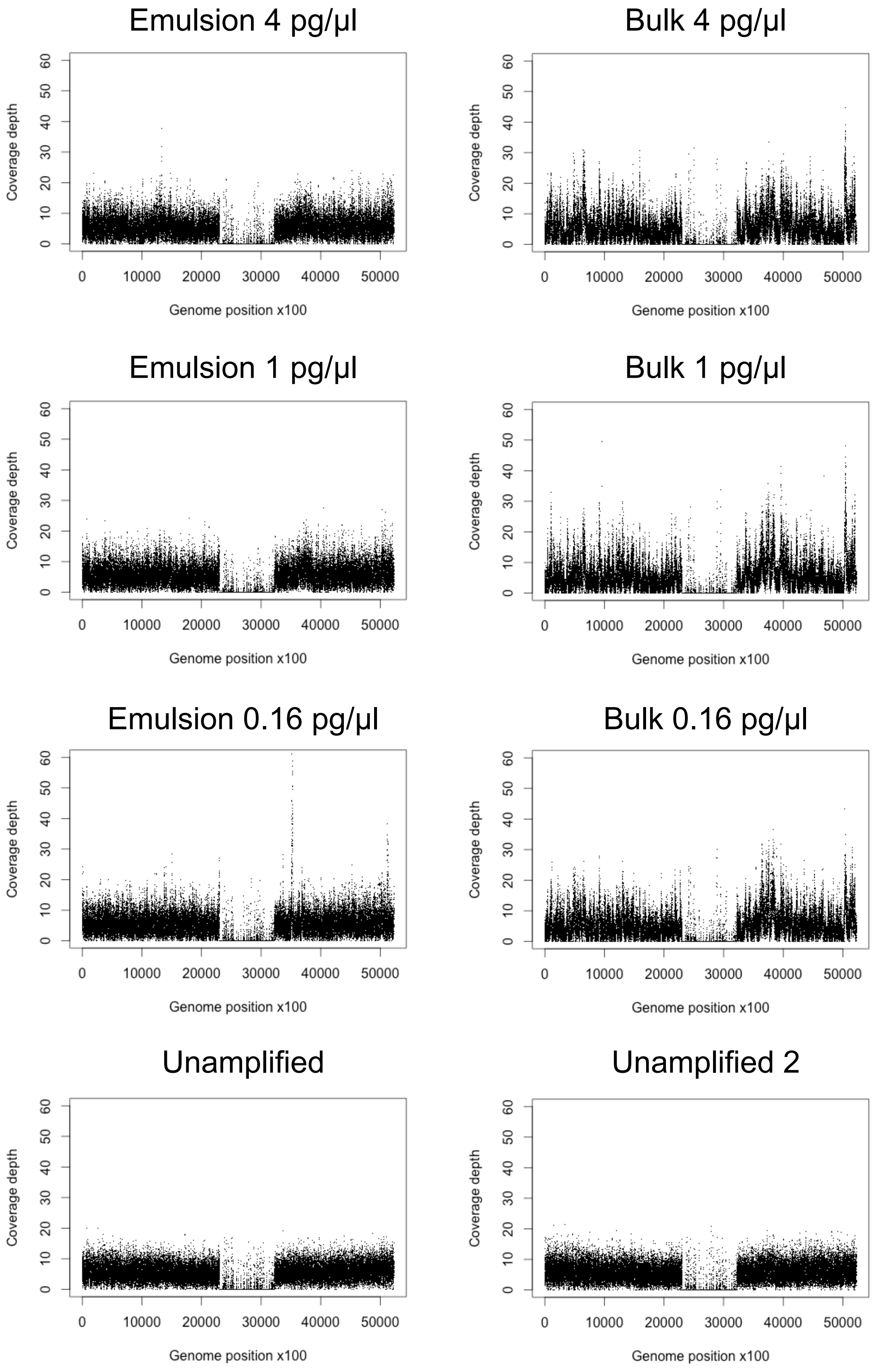

Fig. S3. Coverage depth across *T. roseus* genome. For each sample, reads mapping to the *T. roseus* genome with mapping quality >0 were subsampled to 5x the size of the genome. The mean coverage depth was calculated for 100 bp windows and the average coverage depth for each window was then plotted against its position on the genome.

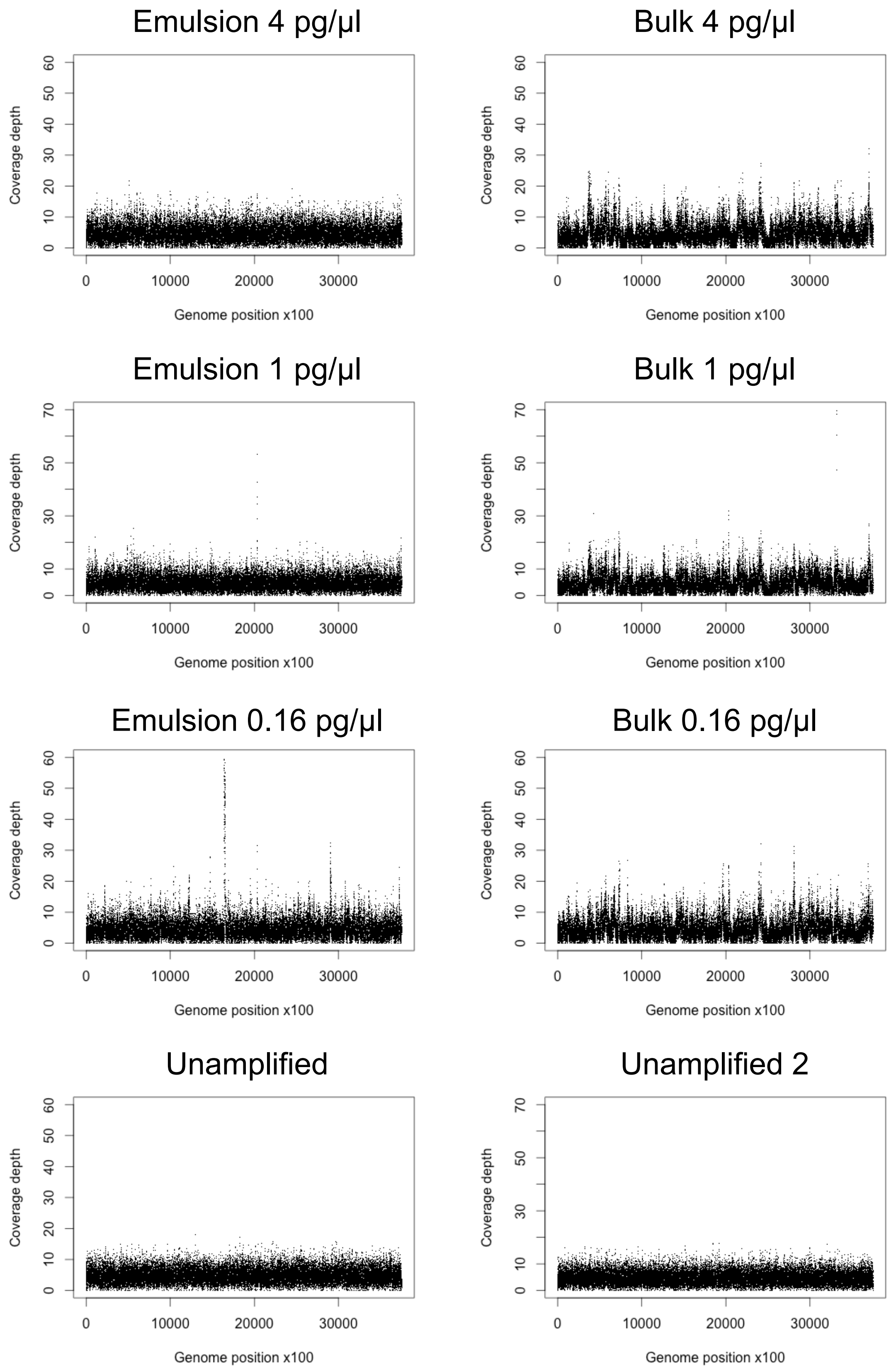

Fig. S4. Coverage depth across *C. akajimensis* genome. For each sample, reads mapping to the *C. akajimensis* genome with mapping quality >0 was subsampled to 5x the size of the genome. The mean coverage depth was calculated for 100 bp windows and the average coverage depth for each window was then plotted against its position on the genome.

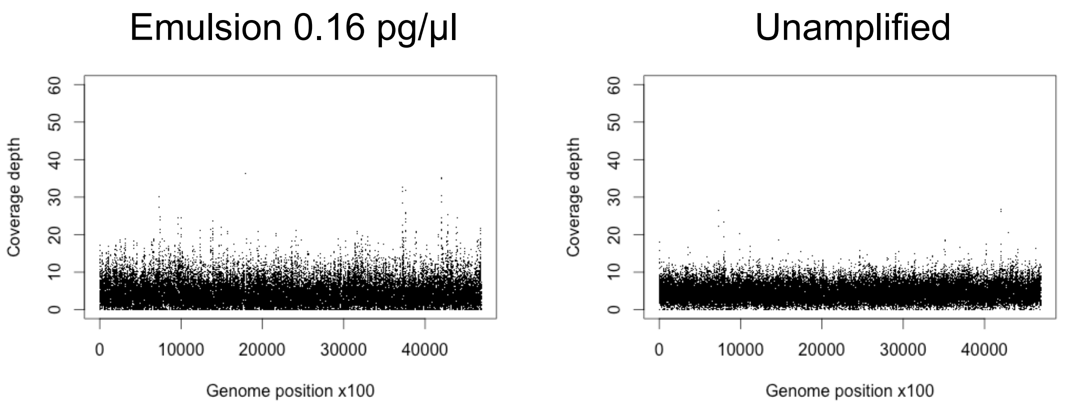

Fig. S5. Coverage depth across *P. stutzeri* genome. For each sample, reads mapping to the *P. stutzeri* genome with mapping quality >0 was subsampled to 5x the size of the genome, when there was enough data. The mean coverage depth was calculated for 100 bp windows and the average coverage depth for each window was then plotted against its position on the genome.

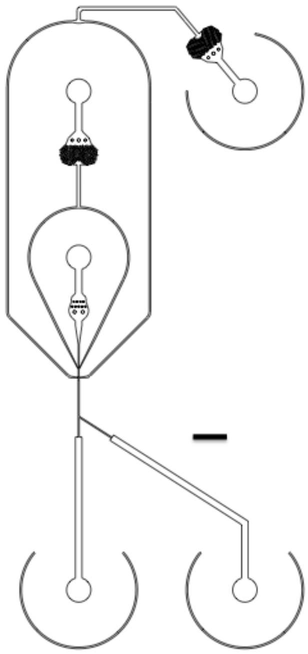

Fig. S6. Design of the microfluidic circuit used for droplet generation. The top inlet to the right was used for oil. The two inlets surrounded by the oil channel were used for the aqueous solutions (MDA reaction mix and denatured DNA). The chip has two alternative outlets (bottom); only one was used for passive collection of emulsion while the other one was plugged. Scale bar represents 1 mm.

Table S1. Genomic DNA pooled for synthetic metagenome

Organism	DSMZ ID	Reference sequence	Genome size (Mb)	GC %	Relative abundance (% of mass)	Relative abundance (% of complete genomes)
Terriglobus roseus	18391	NC_018014.1	5.23	60.3	51.6	45.7
Coraliomargarita akajimensis	45221	NC_014008.1	3.75	53.6	25.8	31.8
Pseudomonas stutzeri	4166	NC_017532.1	4.73	60.6	12.9	12.6
Phaeobacter inhibens	17395	CP002976.1	4.23	59.8	6.5	7.1
Geodermatophilus obscurus	43160	NC_013757.1	5.32	74.0	3.2	2.8

Table S2. Number of sequenced reads for each sample before and after QC trimming

MDA in	MDA input conc. (pg/µl)	Sequenced reads	Reads after QC trimming	GC% of trimmed reads
Emulsion	4	3563062	2480065	57.52%
Emulsion	0.16	2916400	2194067	58.77%
Bulk	4	3881904	2586430	56.76%
Bulk	0.16	4200644	2650793	57.05%
Unamplified	n.a.	1510314	1353968	58.84%
Emulsion	1	1518170	1124831	57.97%
Bulk	1	2333896	1751752	57.47%
Unamplified	n.a.	1455750	1034580	59.02%
Emulsion	0	367010	241869	50.88%
Bulk	0	436900	254157	53.04%

Table S3. Number and percentage of reads mapping to the different species

MDA in	MDA input conc. (pg/µl)	Reads mapping in proper pairs (% of all properly paired reads) *T. roseus*	*C. akajimensis*	*P. stutzeri*	*P. inhibens*	*G. obscurus*
Emulsion	4	782006 (55.9%)	488110 (34.9%)	75062 (5.4%)	24386 (1.7%)	28872 (2.1%)
Emulsion	0.16	693608 (46.7%)	422422 (28.5%)	185868 (12.5%)	89708 (6.0%)	92516 (6.2%)
Bulk	4	732220 (53.7%)	579276 (42.5%)	22026 (1.6%)	10484 (0.8%)	18920 (1.4%)
Bulk	0.16	739348 (59.0%)	465622 (37.2%)	20544 (1.6%)	9454 (0.8%)	18378 (1.5%)
Unamplified	n.a.	622372 (50.5%)	368122 (29.9%)	129766 (10.5%)	49372 (4.0%)	62622 (5.1%)
Emulsion	1	438326 (59.4%)	208816 (28.3%)	54736 (7.4%)	25136 (3.4%)	11002 (1.5%)
Bulk	1	745876 (65.1%)	365238 (31.9%)	15524 (1.4%)	7096 (0.6%)	12508 (1.1%)
Unamplified2	n.a.	412399 (64.0%)	138858 (21.5%)	49010 (7.6%)	21890 (3.4%)	22234 (3.5%)

Table S4. Statistics for reads mapped to all five reference genomes

Sample	No of reads	Amount of data (kb)	Species	Reads mapping in proper pairs (%)[1]	% of genome covered at least 1x	Mean coverage depth	CV%
**Emulsion 4 pg/µl**	598906	128406	*T. roseus*	*320116 (53.45%)*	99.84	13.96	47
			C. akajimensis	*200384 (33.46%)*	99.98	12.86	41
			P. stutzeri	*34457 (5.11%)*	56.44	1.13	141
			P. inhibens	*9946 (1.66%)*	24.38	0.39	271
			G. obscurus	*11954 (2.00%)*	11.17	0.19	497
**Emulsion 0.16 pg/µl**	586014	126598	*T. roseus*	*271150 (46.27%)*	99.79	11.39	56
			C. akajimensis	*164994 (28.16%)*	99.76	10.00	67
			P. stutzeri	*72280 (12.33%)*	87.49	3.03	84
			P. inhibens	*35200 (6.01%)*	70.00	1.83	111
			G. obscurus	*36144 (6.17%)*	49.04	1.05	158
**Bulk 4 pg/µl**	570936	126148	*T. roseus*	*300400 (52.62%)*	99.15	13.11	72
			C. akajimensis	*237332 (41.57%)*	99.90	15.18	55
			P. stutzeri	*8942 (1.57%)*	6.62	0.15	742
			P. inhibens	*4220 (0.74%)*	1.60	0.06	1426
			G. obscurus	*7810 (1.37%)*	1.17	0.07	2149
**Bulk 0.16 pg/µl**	570922	126929	*T. roseus*	*325440 (57.00%)*	99.52	14.41	70
			C. akajimensis	*204756 (35.86%)*	99.85	13.28	57
			P. stutzeri	*9178 (1.61%)*	5.77	0.13	738
			P. inhibens	*4040 (0.71%)*	1.33	0.05	1488
			G. obscurus	*7974 (1.40%)*	1.09	0.06	1410
**Unamplified**	614476	128207	*T. roseus*	*317230 (51.63%)*	99.91	12.51	37
			C. akajimensis	*187732 (30.55%)*	99.97	10.86	35
			P. stutzeri	*66732 (10.86%)*	90.76	2.69	73
			P. inhibens	*25180 (4.10%)*	66.86	1.26	112
			G. obscurus	*31788 (5.17%)*	55.37	0.94	139
**Emulsion 1 pg/µl**	718790	127993	*T. roseus*	416864 (58.00)	99.88	14.49	48
			C. akajimensis	198322 (27.59)	99.92	10.23	47
			P. stutzeri	51954 (7.23)	76.24	1.80	103
			P. inhibens	24224 (3.37)	57.98	1.04	136
			G. obscurus	10454 (1.45)	14.14	0.21	454
**Bulk 1 pg/µl**	617472	128076	*T. roseus*	387978 (62.83)	99.43	16.00	75
			C. akajimensis	189976 (30.77)	99.70	11.64	60
			P. stutzeri	7928 (1.28)	7.39	0.16	711
			P. inhibens	3630 (0.59)	1.72	0.06	1514
			G. obscurus	6420 (1.04)	0.92	0.06	1760
**Unamplified2**	625202	128991	*T. roseus*	412332 (65.95)	99.91	16.15	35
			C. akajimensis	138862 (22.21)	99.83	7.95	43
			P. stutzeri	49008 (7.84)	78.73	1.83	94
			P. inhibens	21878 (3.50)	56.78	1.02	131
			G. obscurus	22234 (3.56)	33.96	0.52	218

Data was subsampled to include, for each sample, the number of reads needed to include the same amount of data for each sample.
[1]Reads mapping in proper pairs are number and percentage of reads as reported by Flagstat.

Table S5. Number of reads and amount of data after subsampling to average 5x coverage of reads mapping to *T. roseus*

MDA in	MDA input conc. (pg/µl)	No of reads	Amount of data (kb)
Emulsion	4	119790	25452
Emulsion	1	146254	25660
Emulsion	0.16	119390	25688
Bulk	4	119930	26086
Bulk	1	128074	26111
Bulk	0.16	113954	24966
Unamplified	n.a.	122160	25254
Unamplified2	n.a.	126764	25963

Table S6. Number of reads and amount of data after subsampling to average 5x coverage of reads mapping to *C. akajimensis*

MDA in	MDA input conc. (pg/µl)	No of reads	Amount of data (kb)
Emulsion	4	82886	18467
Emulsion	1	97876	18392
Emulsion	0.16	80468	18454
Bulk	4	80902	18147
Bulk	1	83880	18090
Bulk	0.16	79012	18125
Unamplified	n.a.	84714	18482
Unamplified2	n.a.	85350	18334

Table S7. Number of reads and amount of data after subsampling to average 5x coverage of reads mapping to *P. stutzeri*

MDA in	MDA input conc. (pg/µl)	No of reads	Amount of data (kb)
Emulsion	4	Not enough	Not enough
Emulsion	1	Not enough	Not enough
Emulsion	0.16	109188	23166
Bulk	4	Not enough	Not enough
Bulk	1	Not enough	Not enough
Bulk	0.16	Not enough	Not enough
Unamplified	n.a.	114954	23312
Unamplified2	n.a.	Not enough	Not enough

Table S8. Basic statistics for *de novo* assemblies

MDA in	MDA input conc. (pg/µl)	Total length	# Contigs	Largest alignment	GC%
Emulsion	1	8506129	891	231380	57.47
Bulk	1	8048554	810	142291	57.31
Unamplified2	n.a.	8831509	767	515551	57.64

Table S9. Basic statistics for mapping contigs from MetaQUAST analysis

Species	MDA in	MDA input conc. (pg/µl)	Total length	Genome fraction (%)	No of contigs >1kb	Largest contig
T. roseus	Emulsion	4	4669339	98.13	277	160737
	Emulsion	0.16	4516159	94.91	431	96006
	Bulk	4	4454035	93.93	480	125031
	Bulk	0.16	4516001	95.08	432	103747
	Unamplified	n.a.	4736972	99.59	114	242057
	Emulsion	1	4651354	97.75	236	231380
	Bulk	1	4503870	94.97	351	142291
	Unamplified2	n.a.	4735468	99.64	89	515551
C. akajimensis	Emulsion	4	3653445	97.36	291	108206
	Emulsion	0.16	3387722	89.90	543	56307
	Bulk	4	3659711	97.58	298	216284
	Bulk	0.16	3580757	95.37	409	104196
	Unamplified	n.a.	3717094	99.45	107	180405
	Emulsion	1	3534432	94.27	451	55069
	Bulk	1	3536302	94.29	455	85353
	Unamplified2	n.a.	3631899	97.03	374	62517
P. stutzeri	Emulsion	4	168436	4.08	105	9730
	Emulsion	0.16	1379962	28.54	728	16450
	Bulk	4	1484	0.14	1	1484
	Bulk	0.16	2827	0.143	2	1493
	Unamplified	n.a.	1436017	29.71	777	49548
	Emulsion	1	366447	7.519	194	24590
	Bulk	1	1846	0.151	1	1846
	Unamplified2	n.a.	428456	9.678	258	18684
P. inhibens	Emulsion	4	3460	0.066	3	1186
	Emulsion	0.16	315314	8.394	204	4063
	Bulk	4	-	-	-	-
	Bulk	0.16	-	-	-	-
	Unamplified	n.a.	174450	5.154	118	5724
	Emulsion	1	25546	1.139	14	7673
	Bulk	1	-	-	-	-
	Unamplified2	n.a.	64557	2.517	46	3028
G. obscurus	Emulsion	4	1625	0.005	1	1625
	Emulsion	0.16	208525	3.311	130	11832
	Bulk	4	-	-	-	-
	Bulk	0.16	-	-	-	-
	Unamplified	n.a.	96135	0.985	56	4597
	Emulsion	1	2911	0.105	2	1574
	Bulk	1	-	-	-	-
	Unamplified2	n.a.	13623	0.121	9	2935
